# Distinct gut bacterial composition in *Anoplophora glabripennis* reared on two host plants

**DOI:** 10.3389/fmicb.2023.1199994

**Published:** 2023-06-19

**Authors:** Xuefei Wang, Hualing Wang, Jianyong Zeng, Zezhao Cui, Shilong Geng, Xiaofei Song, Fengjuan Zhang, Xiaoyu Su, Huiping Li

**Affiliations:** ^1^College of Forestry, Hebei Agricultural University, Baoding, Hebei, China; ^2^Hebei Urban Forest Health Technology Innovation Center, Baoding, Hebei, China; ^3^Key Laboratory of Forest Germplasm Resources and Protection of Hebei Province, Baoding, Hebei, China

**Keywords:** *Anoplophora glabripennis*, intestinal microbial communities, different hosts, 16S rDNA, adaptation

## Abstract

*Anoplophora glabripennis* (Coleoptera: Cerambycidae: Lamiinae) is an invasive wood borer pest that has caused considerable damage to forests. Gut bacteria are of great importance in the biology and ecology of herbivores, especially in growth and adaptation; however, change in the gut bacterial community of this pest feeding on different hosts is largely unknown. In this study, we investigated the gut bacterial communities of *A. glabripennis* larvae fed on different preferred hosts, *Salix matsudana* and *Ulmus pumila*, using 16S rDNA high-throughput sequencing technology. A total of 15 phyla, 25 classes, 65 orders, 114 families, 188 genera, and 170 species were annotated in the gut of *A. glabripennis* larvae fed on *S. matsudana* or *U. pumila* using a 97% similarity cutoff level. The dominant phyla were Firmicutes and Proteobacteria and the core dominant genera were *Enterococcus*, *Gibbsiella*, *Citrobacter*, *Enterobacter*, and *Klebsiella*. There was significantly higher alpha diversity in the *U. pumila* group than in the *S. matsudana* group, and principal co-ordinate analysis showed significant differences in gut bacterial communities between the two groups. The genera with significant abundance differences between the two groups were *Gibbsiella*, *Enterobacter*, *Leuconostoc*, *Rhodobacter*, *TM7a*, *norank*, *Rhodobacter*, and *Aurantisolimonas*, indicating that the abundance of larval gut bacteria was affected by feeding on different hosts. Further network diagrams showed that the complexity of the network structure and the modularity were higher in the *U. pumila* group than in the *S. matsudana* group, suggesting more diverse gut bacteria in the *U. pumila* group. The dominant role of most gut microbiota was related to fermentation and chemoheterotrophy, and specific OTUs positively correlated with different functions were reported. Our study provides an essential resource for the gut bacteria functional study of *A. glabripennis* associated with host diet.

## 1. Introduction

Gut-associated bacteria are vital mediators of plant and insect interactions (Sugio et al., 2015; Berasategui et al., 2017; Bozorov et al., 2019; Chen et al., 2020; Xiong, 2022). Over a long period of coevolution, insect gut bacteria have acquired the ability to quickly adapt to changes in insect diet (Pérez-Cobas et al., 2015; Su et al., 2017), allowing the insect hosts to adapt to different kinds of food resources (Lv et al., 2021; Yuan et al., 2021; Chen et al., 2022). For instance, the gut microbes derived from *Diaphorina citri* (Meng et al., 2022), *Leptinotarsa decemlineata* (Chung et al., 2017), *Phthorimaea operculella* (Zheng et al., 2020), *Henosepilachna vigintioctopunctata* (Lü et al., 2019), and *Curculio chinensis* (Zhang et al., 2020) assist insect hosts in adapting to different host plants by providing nutrients and degrading toxic secondary compounds (Chu et al., 2013; Zhang et al., 2020).

Wood-boring beetles live in a nutrient-poor environment and must cope with large amounts of toxic secondary compounds and plant cell walls mostly comprising macromolecules that are difficult to utilize, such as lignin, cellulose, hemicellulose, and pectin (Mattson, 1980; Geib et al., 2008; Shin et al., 2021). The symbiotic microbial community is involved in insect adaptation to natural stress (Mattson, 1980; Anand et al., 2010; Scully et al., 2013; Ayayee et al., 2014). For example, the gut bacteria of two *Apriona* species contribute to the degradation of plant toxic secondary compounds (Zhang S. K. et al., 2022). *Anoplophora glabripennis* larvae harbor a diversity of bacteria in their gut that have putative roles in nutrient provisioning, lignocellulose metabolism, and allelochemical metabolism (Geib et al., 2009; Mason et al., 2019; Wang et al., 2023).

Urban street tree biodiversity plays a crucial role in the function and stability of urban ecosystems (Jim and Zhang, 2013; Zhang and Jim, 2014). *Salix matsudana* and *Ulmus pumila* are common urban street trees of high ecological value in the city of Changchun, China (Zhang et al., 2015; Yang et al., 2022a). The Asian long-horned beetle (*A. glabripennis*) is a serious pest of *S. matsudana* and *U. pumila* (Haack et al., 2006; Hu et al., 2009; Faccoli and Favaro, 2016; Liu et al., 2016); it is native to China and Korea and is largely polyphagous on woody broadleaf trees (Wang, 2004; Haack et al., 2006; Van der Gaag and Loomans, 2014; Meng et al., 2015). The female adult mainly feeds on bark and branches to provide essential nutrition for laying eggs. Newly hatched larvae first feed on the phloem, and the second instar larvae bore into the xylem and heartwood to feed (Luo et al., 2003). Larval feeding destroys vascular tissue that surrounds the tree, eventually leading to its death, thereby seriously threatening the consolidation of afforestation efforts and affecting the sustainable development of forestry (Nowak et al., 2001; Haack et al., 2010; Zhou et al., 2021). Host plants carry a distinct nutritional formula that promotes diverse gut environments in insects that will further shape the structural and functional variations in the gut bacteria communities (Zhang J. et al., 2022). However, there are no studies regarding gut bacterial communities of *A. glabripennis* when reared on *S. matsudana* and *U. pumila* host plants. In this study, we used bacterial 16S-rRNA sequencing to characterize the structure of the bacterial community in the gut of *A. glabripennis* after feeding on two different hosts and to analyze the potential function of this flora. This study aims to assess the effects of host plants on *A. glabripennis* and to provide a basis for the development of efficient and green control measures for the insect.

## 2. Materials and methods

### 2.1. Sample collection

The *A. glabripennis* larvae feeding in naturally infested *S. matsudana* and *U. pumila* were collected in the field in the city of Baoding, Hebei province, China. In August, trees of the two species were selected, with similar diameters at breast height and degrees of damage. The trunks of the two host trees were split and *A. glabripennis* larvae were removed and, respectively, put into autoclave centrifuge tubes and sealed with absorbent cotton balls. Six replicates were taken for each sample from six different trunks. The larvae were identified as fourth instar according to the head capsule width (Li et al., 2011).

To eliminate the effects of microbial residues on insect epidermis on the diversity of intestinal bacteria, the larvae were soaked in 75% ethanol for 3 min and then washed with sterile water three times before dissecting the gut. During the procedure of dissecting the gut, we first used sterile tweezers to place the larvae on sterile petri dishes (diameter 90 mm) and dissected the whole body under a microscope (LEICA M250C). We then removed the fat inside until the gut was exposed. We finally used sterile tweezers to remove the whole gut of the larvae. The dissected gut was transferred to a 1.5-mL centrifuge tube, frozen with liquid nitrogen, and stored in an ultra-low temperature refrigerator (Li et al., 2021).

### 2.2. High-throughput sequencing and analysis

Six larvae from each host were dissected for gut microbial community investigation. The DNA of gut bacteria was extracted using MagPure Stool DNA KF kit B (Magen, China) according to the manufacturer’s protocol. The V3–V4 variable regions of 16S rDNA were amplified with the primers 341F (5′-ACTCCTACGGGAGGCAGCAG-3′) and 806R (5′-GGACTACHVGGGTWTCTAAT-3′). The PCR cycling conditions were as follows: 94°C for 3 min, 30 cycles of 94°C for 30 s, 56°C for 45 s, 72°C for 45 s, and final extension at 72°C for 10 min. The PCR products were purified with AmpureXP beads and eluted in elution buffer. Libraries were qualified using an Agilent 2100 Bioanalyzer (Agilent, USA). The validated libraries were used for sequencing on an Illumina MiSeq platform (BGI, Shenzhen, China) following the standard Illumina pipelines (Li L. Y. et al., 2022).

### 2.3. Quality control and operational taxonomic unit (OTU) identification

Raw reads were filtered according to the following criteria: (i) overlap longer than 10 bp and a mismatch rate less than 0.02 were removed and (ii) nucleotide sequences with average quality scores lower than 20 were removed using the sliding window trimming approach. Paired-end reads were merged by the Fast Length Adjustment of Short reads program (FLASH, v1.2.11) (Magoc and Salzberg, 2011). Chimeric sequences and chloroplast and mitochondria sequences were identified and removed using UCHIME (Edgar et al., 2011). All effective assembled reads were clustered into OTUs with a cutoff value of 97% using UPARSE software (v7.0.1090) (Edgar, 2013). The OTU representative sequences were constructed and further annotated based on RDP Classifier (version 11.5) against the 16S rRNA database Silva SSU138.1 using a confidence threshold of 0.8 (Quast et al., 2013).

### 2.4. Diversity analysis

Alpha and beta diversity were estimated at the OTU level using MOTHUR (Schloss et al., 2009) and UniFrac (Lozupone et al., 2011), respectively. We used R-forge to compare the results of principal coordinates analysis (PCoA). Permutational MANOVA was conducted to further confirm the observed differences (Clarke, 1993), with *p* < 0.05 considered significant. Statistical analysis of metagenomic profiles (STAMP) was used to assess the differences between OTU groups (Parks et al., 2014; Lü et al., 2019). To understand the relationships among the genera, Spearman’s correlation coefficients were used for network analyses. The network topological properties were calculated using Gephi (Jiang et al., 2017). Functional Annotation of Prokaryotic Taxa (FAPROTAX) was used to predict the function of the microbial community in different samples (Louca et al., 2016). A correlation heatmap was used to analyze OTUs and functional abundance correlation.

## 3. Results

## 3.1. 16S rRNA sequencing data quality analysis

**FIGURE 1:**
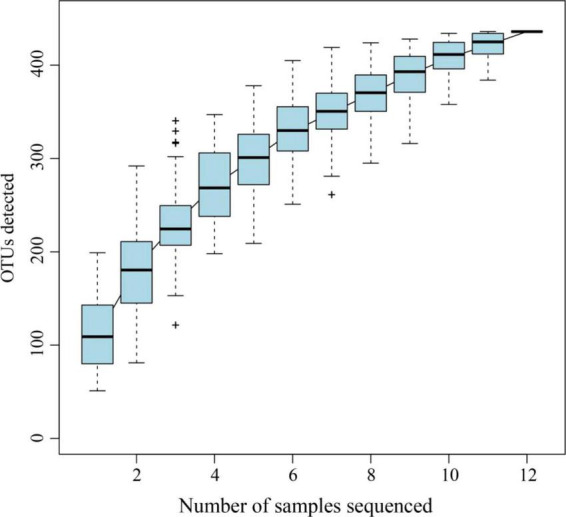
Rarefaction curve of *A. glabripennis* larvae reared on *S. matsudana* and *U. pumila*.

The gut bacterial 16S rRNA sequencing of *A. glabripennis* larvae collected on *S. matsudana* and *U. pumila* resulted in 770,189 clean reads. Each sample contained approximately an average of 64,000 reads, with a range of 59,304–66,418. The range of average sequence length was 425.89–429.86 bp (Table 1). The Good’s coverage of each sample was greater than 0.99, indicating that sequencing analysis of 16S rDNA was representative to a certain extent (Table 1). Rarefaction curves suggested that the data covered most of the microbial information in the sample (Figure 1).

**TABLE 1:** Diversity and abundance of gut bacteria communities of *A. glabripennis* larvae reared on *S. matsudana* and *U. pumila.*

Sample	Sequences	Chao	Richness	Shannon	Simpson	ACE	Evenness	Coverage
*U. pumila_*1	66280	163.83	153	1.96	0.35	166.47	0.27	0.99
*U. pumila_*2	64607	76.5	51	2.01	0.31	72.39	0.35	0.99
*U. pumila_*3	63868	90.5	58	2.05	0.31	103.14	0.35	0.99
*U. pumila_*4	60720	65.13	52	2.47	0.27	65.86	0.43	0.99
*U. pumila_*5	63957	90.69	80	2.16	0.29	96.42	0.34	0.99
*U. pumila_*6	66418	210.25	199	3.18	0.31	202.55	0.42	0.99
*S. matsudana_*1	65864	165.04	143	1.42	0.45	170.83	0.20	0.99
*S. matsudana_*2	65428	156.18	136	1.62	0.51	163.57	0.23	0.99
*S. matsudana_*3	59304	111	104	0.86	0.82	106.95	0.13	0.99
*S. matsudana_*4	61509	194.2	169	3.13	0.18	197.73	0.42	0.99
*S. matsudana_*5	66032	120	113	0.91	0.69	120.19	0.13	0.99
*S. matsudana_*6	66202	117.75	109	0.47	0.89	115.40	0.07	0.99

### 3.2. Composition of *A. glabripennis* gut bacterial community

**FIGURE 2:**
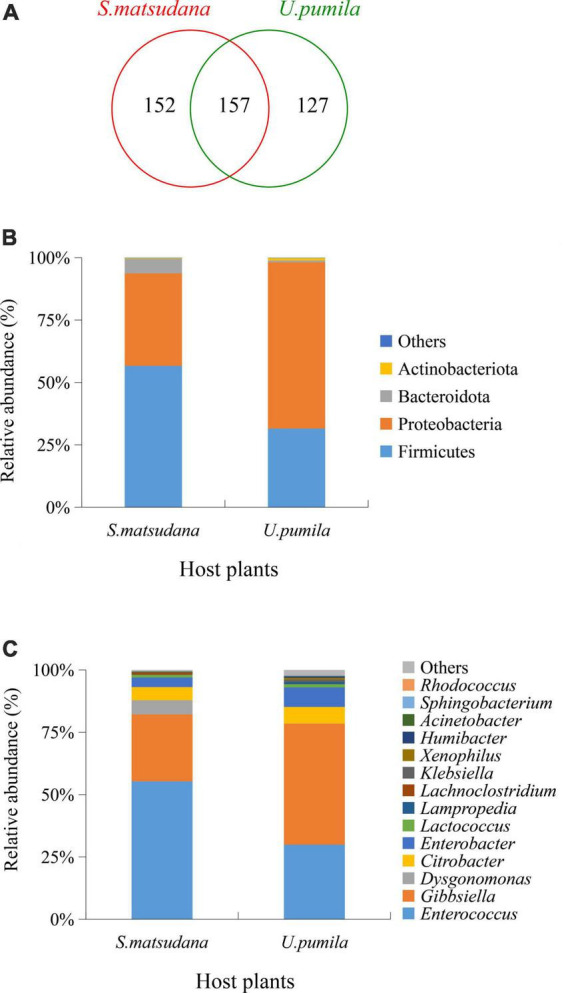
Gut bacteria composition identified in *A. glabripennis* larvae reared on *S. matsudana* and *U. pumila*. Venn diagram represents the number of shared and specific OTUs **(A)**. Relative abundances of bacterial communities at phylum **(B)** and genus **(C)** levels for *A. glabripennis* are illustrated. Columns represent different samples, different colors represent different annotated information, and “others” represents all species except those annotated above.

A total of 436 OTUs were obtained by clustering with 97% similarity, including 15 phyla, 25 classes, 65 orders, 114 families, 188 genera, and 170 species. There were 152 OTUs unique to the gut of the larvae feeding on *S. matsudana* (*S. matsudana* group), 127 OTUs were unique to the gut of larvae feeding on *U. pumila* (*U. pumila* group), and 157 OTUs were shared between those feeding on either host (Figure 2A). At the phylum level, Firmicutes and Proteobacteria were dominant bacteria for both plant hosts, accounting for 93.7% and 98.17% of the total sequences (Figure 2B). At the genus level, the top five OTUs of the *S. matsudana* group were annotated as *Enterococcus* (55.34%), *Gibbsiella* (26.83%), *Dysgonomonas* (5.7%), *Citrobacter* (5.28%), and *Enterobacter* (3.82%), while the top four OTUs of the *U. pumila* group were *Gibbsiella* (48.54%), *Enterococcus* (29.92%), *Enterobacter* (7.84%), and *Citrobacter* (6.67%) (Figure 2C). Five core gut bacteria genera were present in larvae from both hosts: *Enterococcus* (52.83%), *Gibbsiella* (34.01%), *Citrobacter* (7.84%), *Enterobacter* (4.73%), and *Klebsiella* (0.59%) (Figure 2C). Collectively, the results showed that Proteobacteria and Firmicutes were the most abundant phyla for the two hosts, while the dominant genera changed according to the host.

### 3.3. Diversity comparison of gut bacteria

**FIGURE 3:**
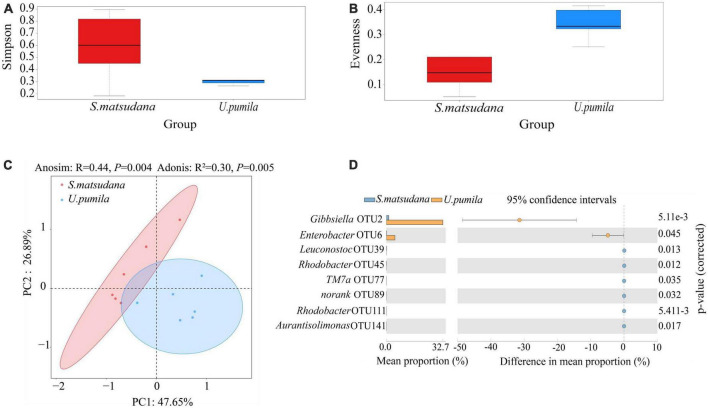
Diversity of the gut bacteria communities of *A. glabripennis* larvae reared on *S. matsudana* and *U. pumila via* Simpson **(A)**, Evenness **(B)**, PCoA **(C)**, and analysis of differences between groups of OTUs **(D)**.

We then compared the diversity of the gut bacterial community derived from the two hosts. The microbial Simpson and Evenness indices of gut bacterial microbiota were significantly higher for the *U. pumila* group than for the *S. matsudana* group (Table 1 and Figures 3A, B). The microbial communities obtained from the different hosts were significantly clustered into two different groups in the PCoA plot (Figure 3C), with all three variables together explaining 74.54% of the total variance (Anosim, *R* = 0.44, *P* = 0.004; Adonis, *R*^2^ = 0.30, *P* = 0.005).

In order to further understand the microbial communities of *A. glabripennis* larvae from the two host plants, the OTUs/species with significant differences were compared (Figure 3D). Eight OTUs were identified with significant differences between the samples from the two groups. The relative abundances of *Gibbsiella* (OTU2) and *Enterobacter* (OTU6 were greater for the *U. pumila* group than for the *S. matsudana* group. The relative abundances of *Leuconostoc* (OTU39), *Rhodobacter* (OTU45), *TM7a* (OTU77), *norank* (OTU89), *Rhodobacter* (OTU111), and *Aurantisolimonas* (OTU141) were higher for the *S. matsudana* group than for the *U. pumila* group. Thus, the host plants influenced the intestinal microbial community of *A. glabripennis* larvae.

### 3.4. Construction of ecological network of intestinal flora

**FIGURE 4:**
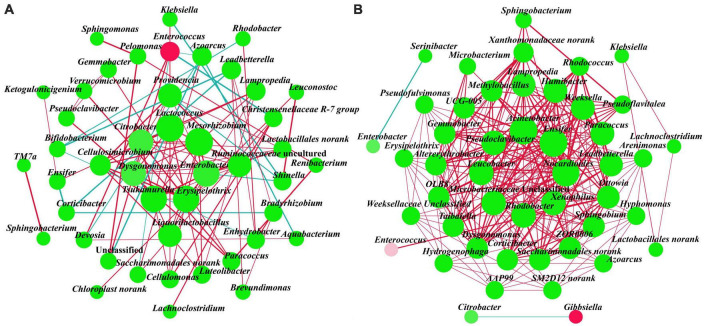
Interaction networks of gut bacteria genera from the *S. matsudana* **(A)** and *U. pumila* **(B)** groups based on correlation analysis. For each panel, the node represents unique genera, the size of each node represents degree, the red line represents positive correlation, and the blue line represents negative correlation. When a correlation coefficient exceeds 0.6 and *p* < 0.05, the relationships are kept.

Two association networks were constructed to determine the patterns of gut bacterial communities of *A. glabripennis* fed with *S. matsudana* and *U. pumila* (Figures 4A, B). The network diagram for the *S. matsudana* group included 45 nodes and 100 edges (85 positive and 15 negative correlations) and the *U. pumila* group included 47 nodes and 262 edges (260 positive and two negative correlations). This indicated that the complexity of the network structure and the modularity were higher in the *U. pumila* group than in the *S. matsudana* group. We found that in the *S. matsudana* group, the degrees of *Lactococcus* and *Mesorhizobium* were 11; *Enterobacter*, *Erysipelothrix*, and *Tsukamurella* were 10; and *Dysgonomonas* and *Citrobacter* were 9, and there were positive correlations among them. Degrees equal to or larger than 18 in *U. pumila* were as follows: *Acinetobacter* and *Ensifer* with 23, *Nocardioides* with 22 and *Xenophilus*, *Rhodobacter*, *Microbacteriaceae_*Un classified, *Leucobacter*, and *Pseudoclavibacter*. *Xenophilus, Rhodobacter, Microbacteriaceae*_Unclassified, *Leucobacter*, and *Pseudoclavibacter* with 18. There were positive correlations among these genera. Overall, the results indicated that there were more cooperation and exchange events among most bacterial genera during the adaptation of *A. glabripennis* larvae to different hosts.

### 3.5. Functional prediction of gut bacteria

**FIGURE 5:**
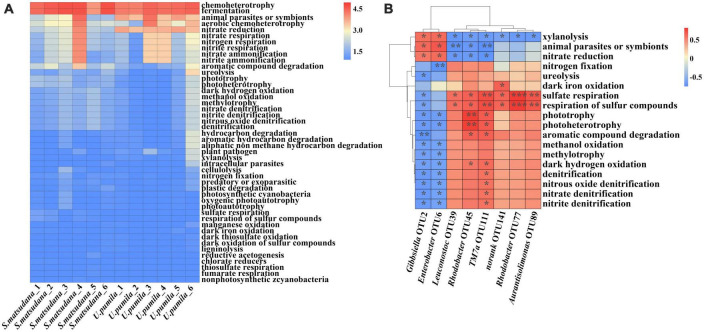
Functional predictions **(A)** and the relationship between OTUs and functional abundance **(B)** of the gut bacteria communities of *A. glabripennis* larvae reared on *S. matsudana* and *U. pumila*. The color scale represents the normalized values of relative abundances by log_10_ **(A)**, blue indicates negative correlation, red indicates positive correlation, and a darker color indicates a stronger correlation (*p < 0.05, **p < 0.01, ***p < 0.001) **(B)**.

Comparative functional analysis showed similar function patterns among bacterial communities of the two host plants. A total of 46 putative functions were identified from the two host plants. Most OTUs were annotated with fermentation and chemoheterotrophy functions. The other eight main functions were nitrate ammonification, nitrite ammonification, nitrite respiration, nitrate respiration, nitrogen respiration, nitrate reduction, aromatic compound degradation, and aerobic chemoheterotrophy (Figure 5A). To further investigate the relationship between OTUs and functions, the functions with significance that were correlated with specific OTUs were analyzed (Figure 5B). *Gibbsiella* (OTU2) and *Enterobacter* (OTU6) were positively correlated with nitrate reduction animal parasites and symbiots and xylanolysis, and the relative abundance of these two OTUs was greater for the *U. pumila* group than for the *S. matsudana* group. *Leuconostoc* (OTU39), *Rhodobacter* (OTU45), *TM7a* (OTU77), *norank* (OTU89), *Rhodobacter* (OTU111), and *Aurantisolimonas* (OTU141) were positively correlated with sulfate respiration and respiration of sulfur compounds. Additionally, *Rhodobacter* (OTU45) was positively correlated with aromatic compound degradation, dark hydrogen oxidation, phototrophy and photoheterotrophy. *Aurantisolimonas* (OTU141) was positively correlated with dark iron oxidation. *Rhodobacter* (OTU111) was positively correlated with nitrate denitrification, nitrite denitrification, nitrous oxide denitrification, denitrification, dark hydrogen oxidation, aromatic compound degradation, phototrophy and photoheterotrophy. The relative abundances of *Leuconostoc* (OTU39), *Rhodobacter* (OTU45), *TM7a* (OTU77), *norank* (OTU89), *Rhodobacter* (OTU111), and *Aurantisolimonas* (OTU141) were higher for the *S. matsudana* group than for the *U. pumila* group. Overall, the dominant roles of most gut bacteria were related to fermentation and chemoheterotrophy, and OTUs that were positively correlated with different functions were identified.

## 4. Discussion

Gut bacteria in insects play important and diverse roles in host digestion (Jing et al., 2020), detoxification (Blanton and Peterson, 2020; Siddiqui et al., 2022), development (Duplais et al., 2021), pathogen resistance (Khan et al., 2021), immune response (Engel and Moran, 2013), and the production of essential vitamins and amino acids (Bisch et al., 2018). In contrast, the bacterial diversity, structure, or composition of the gut in many insects (Montagna et al., 2015; Li et al., 2021) could be influenced by host diets. In this study, the composition and diversity of the gut bacteria of *A. glabripennis* from two hosts were studied. The bacterial Simpson and Evenness indices (alpha diversity) were higher for *U. pumila* larvae than *S. matsudana* larvae, and beta diversity significantly differed between the two hosts. The abundance of *Enterococcus* was higher in the *S. matsudana* group than in the *U. pumila* group, while *Gibbsiella* was more abundant in the *U. pumila* group than in the *S. matsudana* group. Consistent with previous findings in *Spodoptera frugiperda* (Jones et al., 2019), *Grapholita molesta* (Liu et al., 2020), and *Cnaphalocrocis medinalis* (Yang et al., 2022b) that fed on different food sources, we found high variability in gut bacterial composition and abundance. Moreover, the observed variations in the gut of *A. glabripennis* larvae could be attributed to the diverse secondary metabolites of the different plants or the different compositions of endophytic bacteria in plants (Afzala et al., 2019; Guerrieri et al., 2019).

Our findings revealed that the bacterial communities of *A. glabripennis* larvae fed on *S. matsudana* and *U. pumila* were similar at the phylum level, consisting of Proteobacteria and Firmicutes, which is consistent with the results of Schloss et al. (2006), who identified the same two abundant bacteria phyla in *A. glabripennis*. Similar to Wang et al. (2022), who identified that genera *Enterococcus* and *Gibbsiella* were dominant in *A. glabripennis* larvae fed on *Populus gansuensis*, we found that the same genera were abundant in gut bacteria of *A. glabripennis*. *Enterococcus* was recognized as promoting the growth and development of host insects by synthesizing vitamins and amino acids (Sabo et al., 2020; Li C. M. et al., 2022), improving gut immunity (Ennahar et al., 1998; Mohamed and Huang, 2007; Ruiz-Rodriguez et al., 2012; Shao et al., 2017) and degrading carbohydrates and secondary compounds (Dantur et al., 2015; Vilanova et al., 2016; Li C. M. et al., 2022). Thus, we speculated that *Enterococcus* contributes to the adaptation of wood borer beetles to different hosts. *Gibbsiella* can degrade lignocellulosic compounds and fix nitrogen (Rizzi et al., 2013). Interestingly, *Gibbsiella* is present in many wood-boring beetles, such as *Apriona germari* and *Apriona swainsoni* (Zhang J. et al., 2022; Zhang S. K. et al., 2022), *Agrilus biguttatus* (Brown et al., 2015), and *Anoplophora chinensis* (Rizzi et al., 2013), suggesting that *Gibbsiella* may play an important role in the adaptation of wood-boring insects to different food sources.

The association network revealed that different hosts influenced the bacterial networks. The higher numbers of network topology properties, such as the number of nodes, positive correlations, negative correlations, and degree observed in the *U. pumila* group indicated a complex network for this group. This further suggests that compared with *S. matsudana*, the *U. pumila* group may be superior at enhancing gut bacterial network complexity and systemic resistance to the external environment. We also found that there were cooperation and exchange events among most bacterial groups. For example, *Dysgonomonas* and *Citrobacter* showed positive correlations in the *S. matsudana* group and have been reported to possess the ability to degrade cellulose (Li X. Y. et al., 2022), indicating that these two genera might work together to improve degradation efficiency. In the *U. pumila* group, *Acinetobacter* and *Rhodobacter* showed positive correlations, and the former is capable of degrading cellulose and hydrocarbons (Pourramezan et al., 2012; Rehman et al., 2018), while the latter can fix nitrogen (Liu et al., 2018; Wang et al., 2019; Xu et al., 2022). In the treatment of rural wastewater in Dianchi Lake, *Rhodobacter* contributed to biofilm formation and the degradation of pollutants in the early stage of adding microbial carriers, and *Acinetobacter* contributed to nitrogen removal in the stable stage of the microbial community (Chen et al., 2019). Thus, it seems that there is a cooperative relationship between these two genera. However, there is also competition among gut bacteria (Umu et al., 2017; Cantu-Jungles and Hamaker, 2020). We found a negative correlation between *Enterococcus* and *Christensenellaceae R-7 group* in the *S. matsudana* group, which is consistent with the results of Zhang et al. (2019), who found that the abundance of *Christensenellaceae R-7 group* in the gut decreased after *Enterococcus faecalis* was added into the diet of Hy-Line Brown laying hens.

There were 46 functions of gut bacteria predicted and shared between the two hosts. Consistent with the findings for *Nilaparvata lugens* (Wang et al., 2021), *Apriona germari* (Zhang J. et al., 2022), and *A. glabripennis* (Wang et al., 2023), fermentation and chemoheterotrophy functions were annotated as the main functions. These two functions were mainly contributed by the abundance of Proteobacteria and Firmicutes (Ugwu et al., 2022). Similarly, *Enterobacter*, *Klebsiella*, and *Citrobacter* species (belonging to Proteobacteria) are capable of fermentation (Ogilvie et al., 1997; Dong et al., 2020). In addition, *Gibbsiella*, also a member of Proteobacteria, can ferment glucose (Campos et al., 2014), while glucose can induce lipid accumulation (Li et al., 2015). This indicates that *Gibbsiella* might participate in fermenting glucose into lipids that further promote the growth and development of *A. glabripennis* larvae. *Citrobacter* and *Klebsiella* can degrade aromatic compounds (Ammar et al., 2005; Kraigher and Mandic-Mulec, 2020; Zhou et al., 2022). Some functions of gut bacteria are related to the nitrogen cycle, which is essential for insect growth and development. *Enterobacter*, *Klebsiella*, and *Citrobacter* are capable of nitrate reduction and/or nitrate respiration (Ogilvie et al., 1997; Dong et al., 2020; Jiang et al., 2022). *Citrobacter* is commonly associated with denitrification processes (Meng et al., 2019). Furthermore, *Enterobacter*, *Klebsiella*, and *Citrobacter* have been identified as being associated with detoxification (Zhang et al., 2013; Cheng et al., 2017; Francoeur et al., 2020; Li Z. et al., 2022). The relationship of OTUs and functional reanalysis showed that *Enterobacter* (OTU6) was related to nitrate reduction and xylanolysis, which is consistent with the results of Zhao et al. (2020) and Xia (2014). *Leuconostoc* (OTU39), *Rhodobacter* (OTU45), *TM7a* (OTU77), *norank* (OTU89), *Rhodobacter* (OTU111), and *Aurantisolimonas* (OTU141) were predicted to be positively correlated with nitrogen, sulfur, and hydrogen cycles, indicating that these OTUs were closely related to *A. glabripennis* growth and development. However, one problem with this study is that all outcomes were from bioinformatic predictions, and the functions of the detected OTUs need to be verified in the future.

## 5. Conclusion

This study demonstrates that (1) the gut bacterial communities of larvae that fed on *S. matsudana* differed from those that fed on *U. pumila*, (2) there were many events of cooperation and communication among gut bacteria, and (3) OTUs that positively correlated with different functions in the adaptation to different hosts were identified. Our study significantly contributes to the understanding of the relationship between gut microbes of *A. glabripennis* and insect–host plant interactions.

## Data availability statement

The datasets presented in this study can be found in online repositories. The names of the repository/repositories and accession number(s) can be found below: https://www.ncbi.nlm.nih.gov/, PRJNA949278 https://www.ncbi.nlm.nih.gov/, SUB12998666.

## Author contributions

HL designed the project. XW, HW, and JZ analyzed the data and wrote the manuscript. XW, XS, and ZC performed the experiments. SG, XS, and FZ embellished the picture. All authors contributed to the article and approved the submitted version.
